# Supplementary material for: Molecular evidence of widespread benzimidazole drug resistance in Ancylostoma caninum from domestic dogs throughout the USA and discovery of a novel β-tubulin benzimidazole resistance mutation
Source: PLoS Pathog. 2023 Mar 2;19(3):e1011146. doi: 10.1371/journal.ppat.1011146 (PMC10013918; doi:10.1371/journal.ppat.1011146)
Supplement: S1 Appendix — (DOCX) [file ppat.1011146.s001.docx]

***A. caninum* pooled samples across the USA.**

The hookworm positive fecal samples collected from dogs across the USA were classified as “many”, “moderate”, “few”, and “rare” based on IDEXX’s semi-quantitative classification. Samples classified as “rare”, as well as those classified as “few” but with <1g of feces available, were combined into pools before isolating the eggs. 65 such pooled samples, representing a total of 357 dogs across the USA, were sequenced at depth using the Illumina Miseq platform to enable the molecular detection of benzimidazole resistance from samples with low egg counts. Of these 65 pooled samples, the 293 bp and the 340 bp isotope-1 β-tubulin fragments encompassing codons 134, 167, 198, and 200 were successfully amplified from 63 samples. The samples were sequenced and analyzed using the same methods as described for the individual samples. The F167Y(TTC>TAC) and the novel Q134H(CAA>CAT) resistance mutations were present in 77.7% (49/63) and 53.9% (34/63) of the pooled samples, respectively. Their overall frequencies in the positive samples were 33.1% (95% C.I. 25.0% - 41.3%) and 10.2% (95% C.I. 5.3% - 15.1%), respectively (S6A and 6B Figs). Variant calling at codons 198 and 200 revealed that 100% of the pooled samples contained susceptible alleles at these codons (S8 Fig).
