# Supplementary figures and images for: Molecular evidence of widespread benzimidazole drug resistance in Ancylostoma caninum from domestic dogs throughout the USA and discovery of a novel β-tubulin benzimidazole resistance mutation
Source: PLoS Pathog. 2023 Mar 2;19(3):e1011146. doi: 10.1371/journal.ppat.1011146 (PMC10013918; doi:10.1371/journal.ppat.1011146)

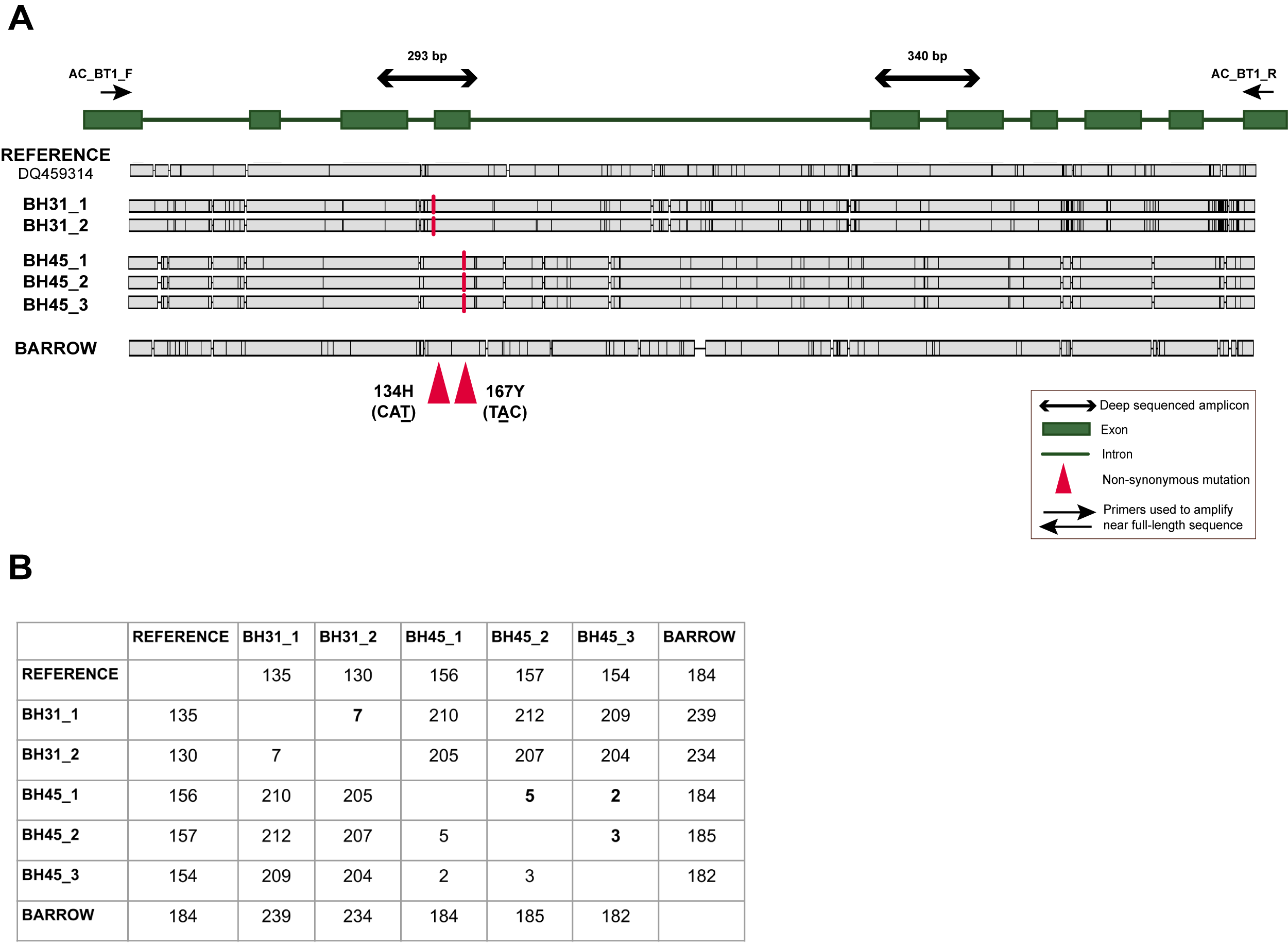

Supplement: S1 Fig — (A): Multiple Sequence Alignment of the 3,467 bp long A. caninum isotype-1 β-tubulin near full-length genomic sequence cloned and sequenced from greyhound A. caninum samples BH31 and BH45, the benzimidazole-susceptible lab sample (Barrow), and the A. caninum isotype-1 β-tubulin reference sequence (Genbank accession: DQ459314). Following Sanger sequencing, the sequences of each clone were assembled de novo using the Geneious software and the assembled consensus sequences for each clone were aligned using the MUSCLE tool for multiple sequence alignment in Geneious v. 10.0.9. The synonymous and non-synonymous polymorphisms relative to the reference sequence, DQ459314, are highlighted in black and red, respectively. (B): Distance matrix showing the pairwise number of nucleotide differences among the sequences. (TIF) [file ppat.1011146.s002.tif]

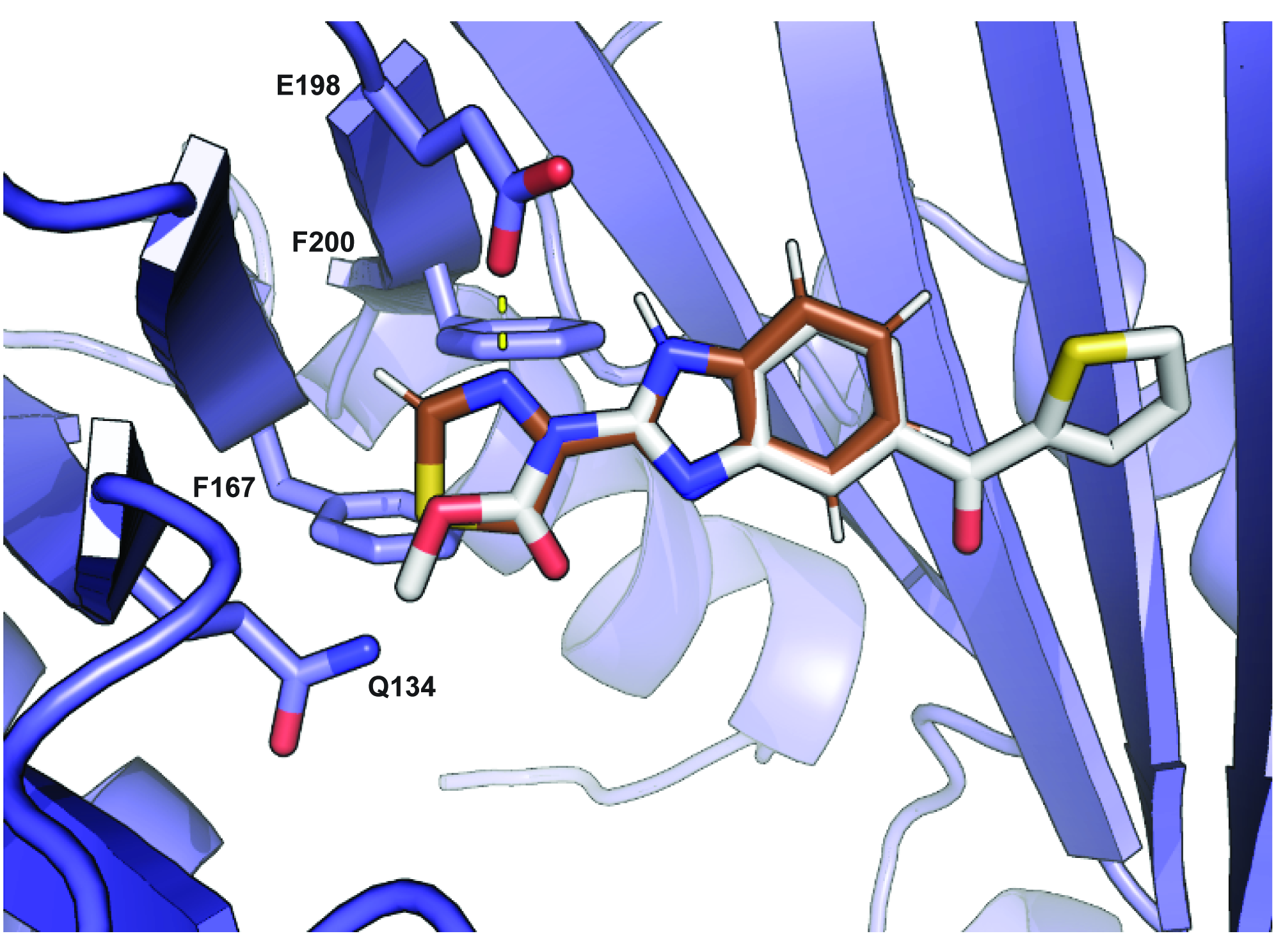

Supplement: S2 Fig — Structural model for A. caninum isotype-1 β-tubulin made using AlphaFold2 (PMID: 34265844) (purple) aligned to Porcine β-tubulin (not shown) bound to nocodazole (white) (from PDB 5CA1) with thiabendazole modeled (brown). Potential interaction with residue 198 shown (dashed lines) with other features occupying the similar volume in the pocket to the methyl ester terminus. (TIF) [file ppat.1011146.s003.tif]

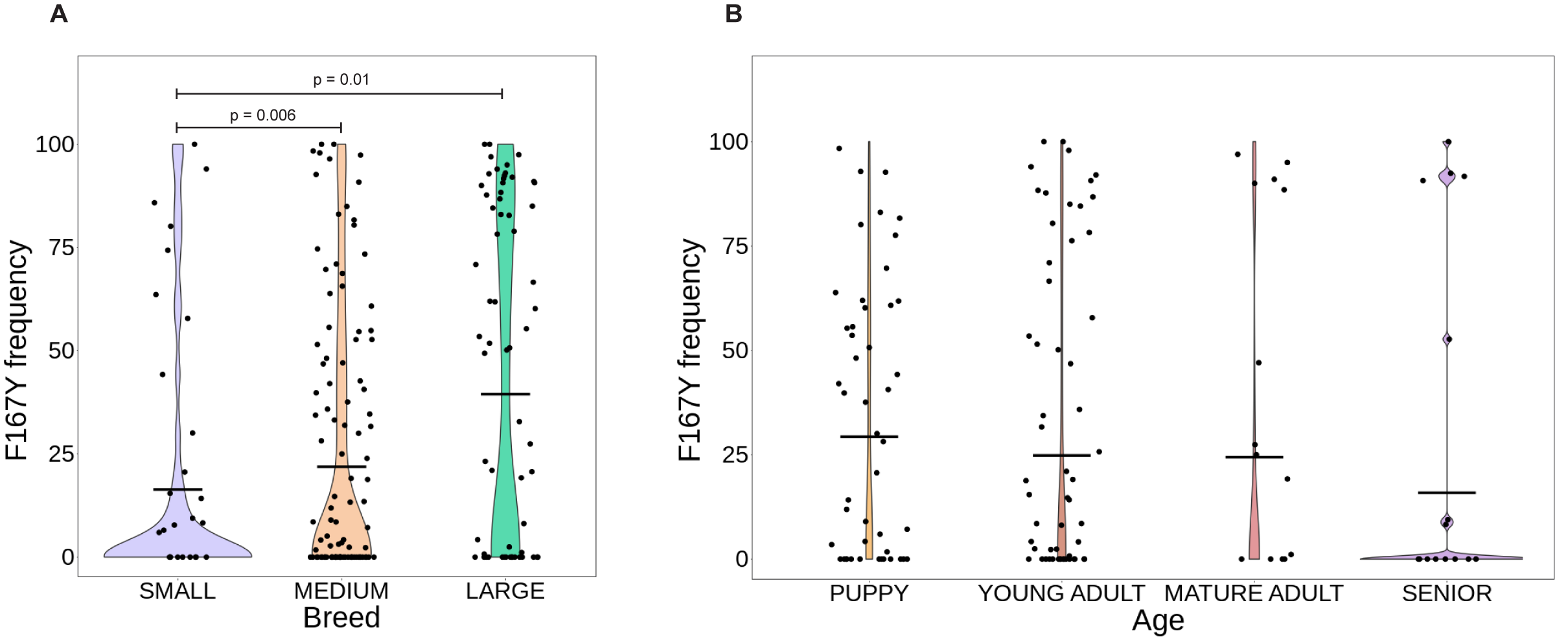

Supplement: S3 Fig — (A) Violin Plot of the 167Y(TAC) resistance allele frequencies in A. caninum from dogs of different breed sizes. The mean frequency is indicated by a horizontal line and any statistically significant differences calculated using the pairwise Wilcoxon rank sum test (p<0.05) between the regions are indicated (p-value >0.05 not indicated). (B) Violin Plot of the 167Y(TAC) resistance allele frequencies in A. caninum from dogs of different age groups. The mean frequency is indicated by a horizontal line and any statistically significant differences calculated using the pairwise Wilcoxon rank sum test (p<0.05) between the regions are indicated (p-value >0.05 not indicated). (TIF) [file ppat.1011146.s004.tif]

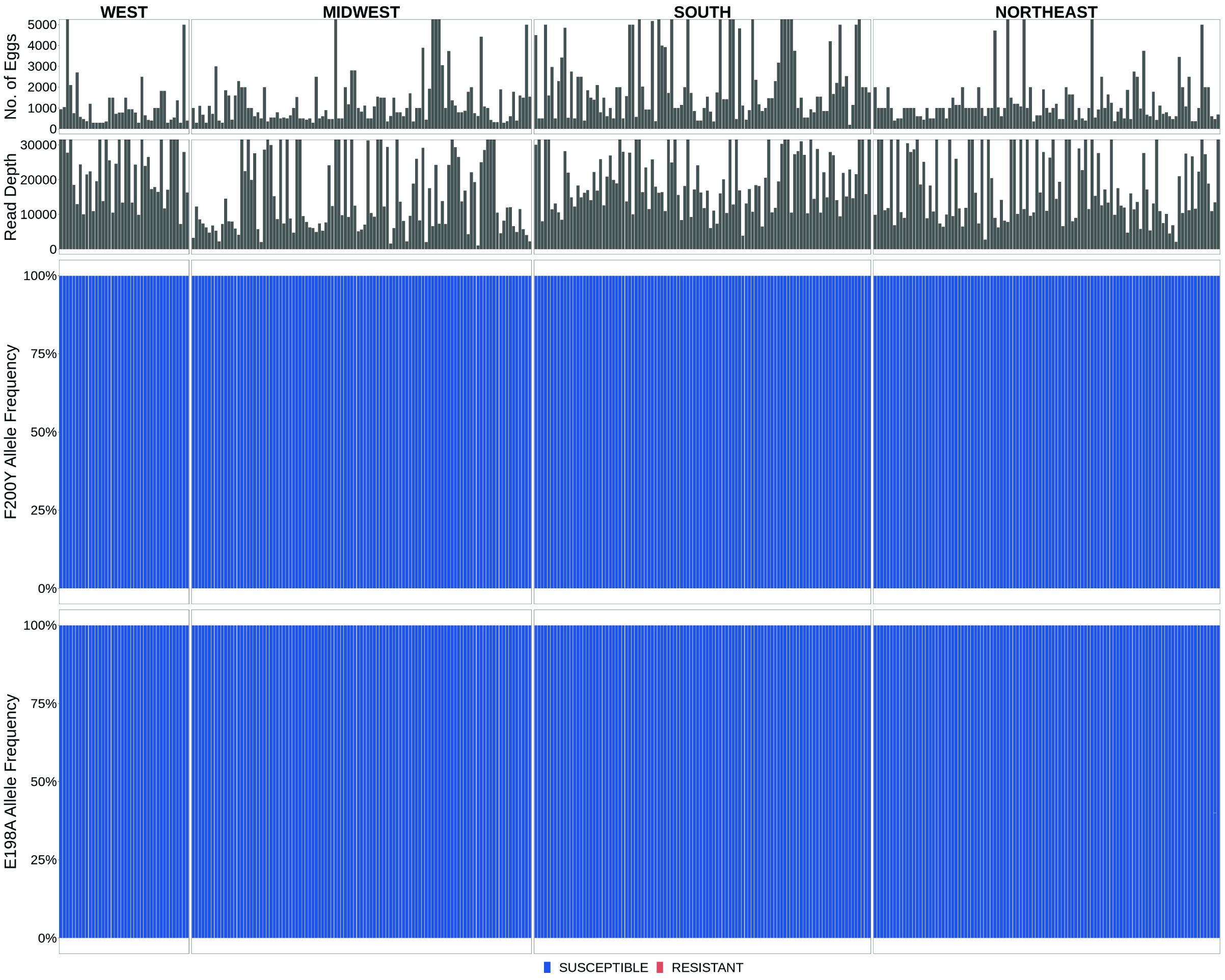

Supplement: S4 Fig — Deep amplicon sequencing data of the 340 bp fragment of A. caninum from the 312/ 328 individual samples across the USA. The top panel is a bar chart showing the number of eggs used to make each genomic DNA preparation, in the middle is a bar chart showing the mapped read depth for each sample, and the lower charts show the frequency of resistance or susceptible alleles present at codons 198 and 200 of the isotype-1 β-tubulin gene. Blue bars indicate the susceptible alleles 198E(GAA) and 200F(TTC) at both the codons. (TIF) [file ppat.1011146.s005.tif]

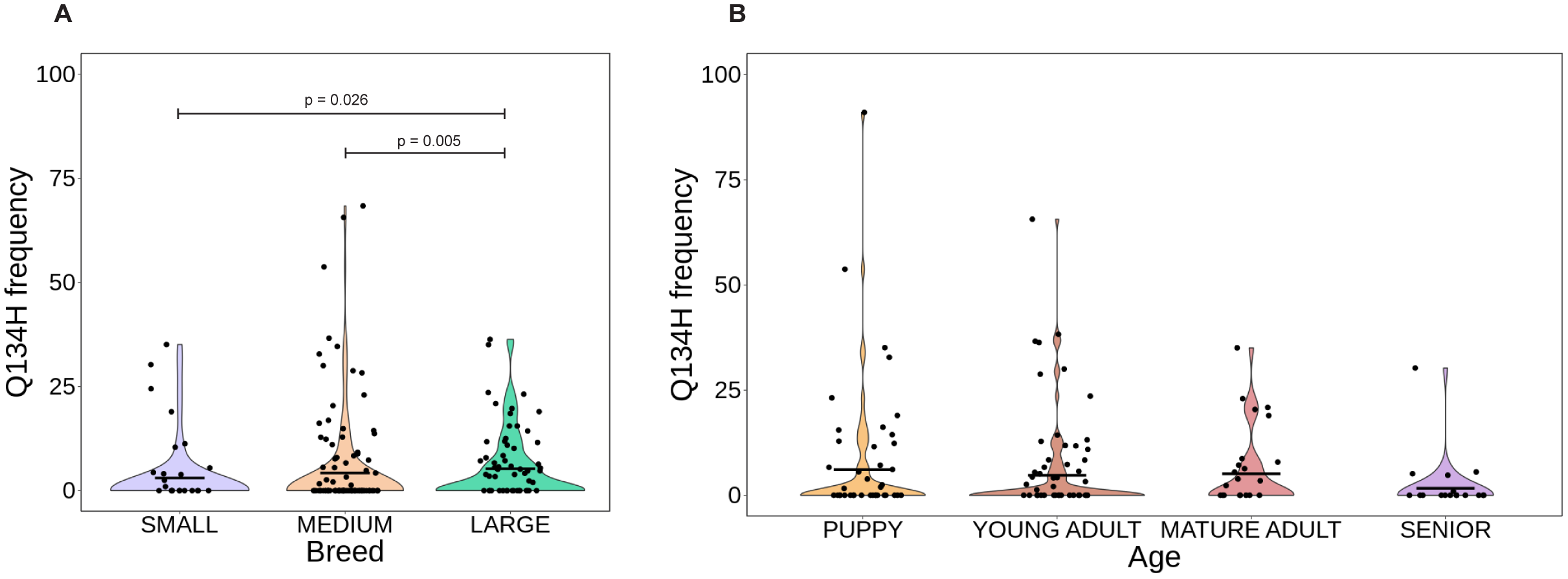

Supplement: S5 Fig — (A) Violin Plot of the 134H(CAT) resistance allele frequencies in A. caninum from dogs of different breed sizes. The mean frequency is indicated by a horizontal line and any statistically significant differences calculated using the pairwise Wilcoxon rank sum test (p<0.05) between the regions are indicated (p-value >0.05 not indicated). (B) Violin Plot of the 134H(CAT) resistance allele frequencies in A. caninum from dogs of different age groups. The mean frequency is indicated by a horizontal line and any statistically significant differences calculated using the pairwise Wilcoxon rank sum test (p<0.05) between the regions are indicated (p-value >0.05 not indicated). (TIF) [file ppat.1011146.s006.tif]

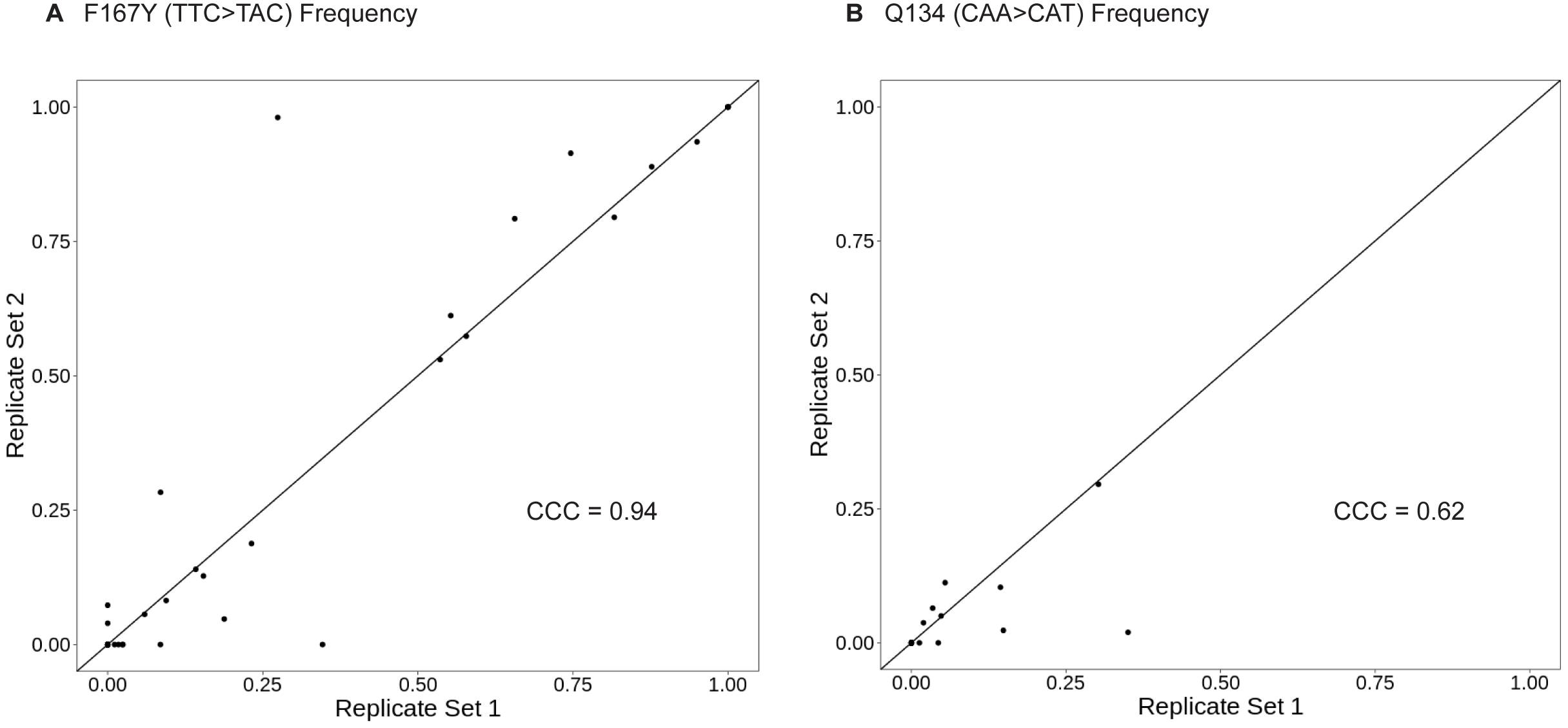

Supplement: S6 Fig — (A): Scatterplot comparing the 167Y(TAC) resistance mutation frequencies determined from two independent replicate PCRs and Miseq runs for 50 samples. 24/50 samples carried the resistance mutation. The solid diagonal line indicates perfect agreement between the two runs. Lin’s Concordance Correlation Coefficient (CCC) indicates the level of agreement between the two sets of replicates. (B): Scatterplot comparing the 134H(CAT) resistance mutation frequencies determined from two independent replicate PCRs and Miseq runs for 50 samples. 10/50 samples carried the resistance mutation. The solid diagonal line indicates perfect agreement between the two runs. Lin’s Concordance Correlation Coefficient (CCC) indicates the level of agreement between the two sets of replicates. (TIF) [file ppat.1011146.s007.tif]

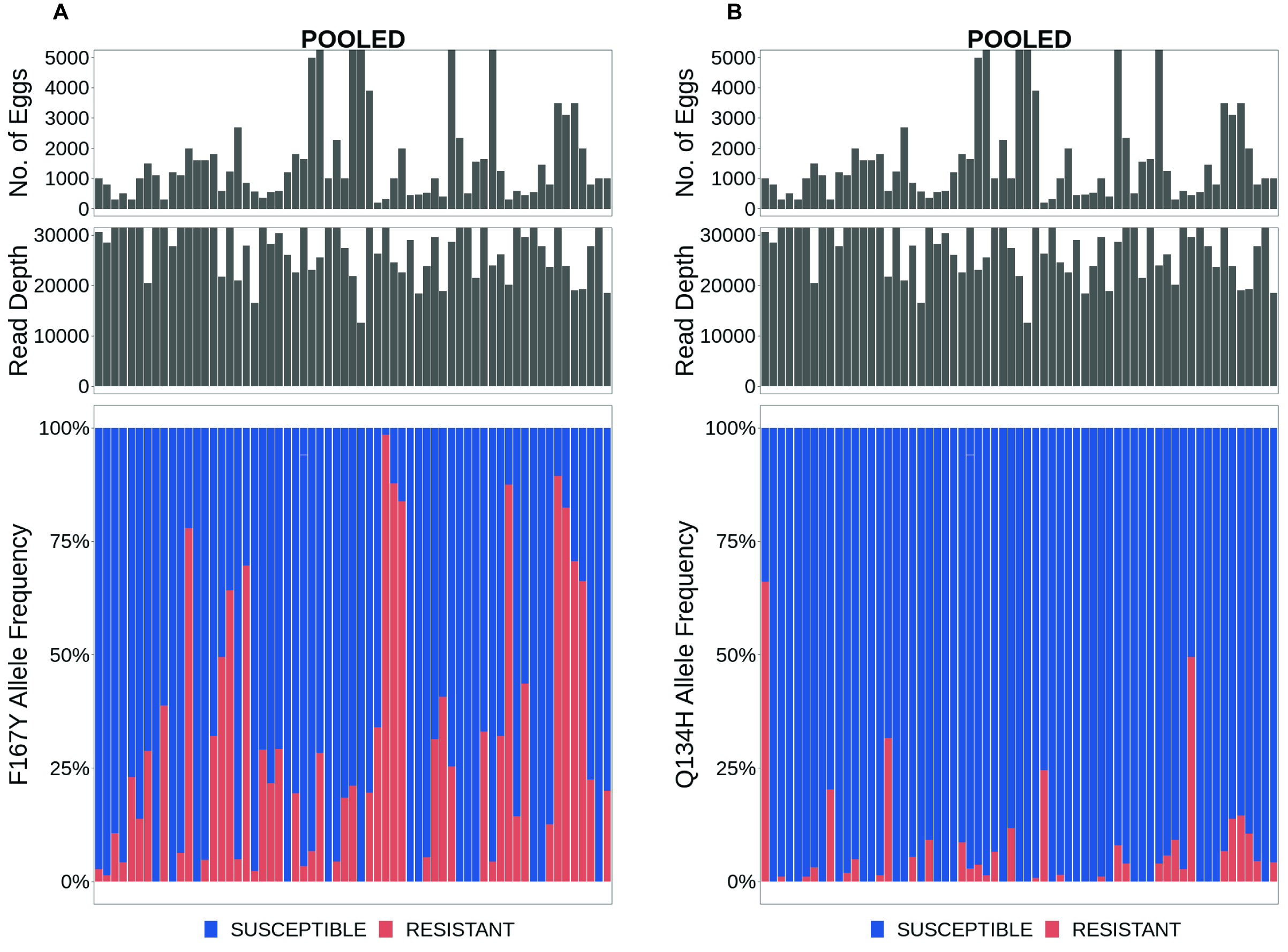

Supplement: S7 Fig — (A): Deep amplicon sequencing data of the 293 bp isotype-1 β-tubulin fragment of A. caninum from the 63/ 65 pooled samples across the USA. The top panel is a bar chart showing the number of eggs used to make each genomic DNA preparation, in the middle is a bar chart showing the mapped read depth for each sample, and the lower chart shows the relative frequency of the F167Y(TTC>TAC) mutation. Red bars indicate the 167Y(TAC) resistance allele and the blue bars indicate the 167F(TTC) susceptible allele. (B): Deep amplicon sequencing data of the 293 bp isotype-1 β-tubulin fragment of A. caninum from the 63/ 65 pooled samples across the USA. The top panel is a bar chart showing the number of eggs used to make each genomic DNA preparation, in the middle is a bar chart showing the mapped read depth for each sample, and the lower chart shows the relative frequency of the Q134H(CAA>CAT) mutation. Red bars indicate the 134H(CAT) resistance allele and the blue bars indicate the 134Q(CAA) susceptible allele. (TIF) [file ppat.1011146.s008.tif]

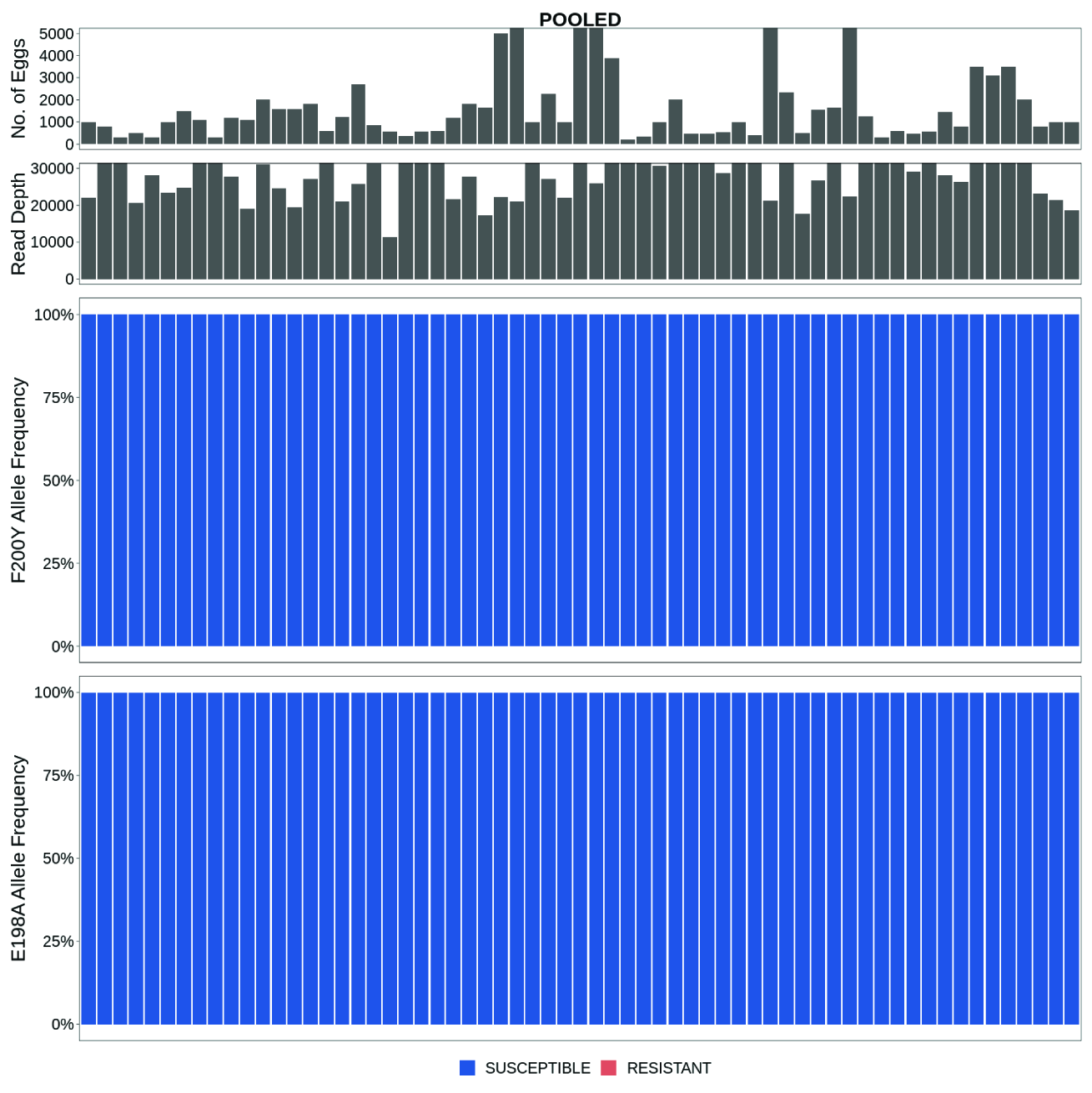

Supplement: S8 Fig — Deep amplicon sequencing data of the 340 bp fragment of A. caninum from the 63 pooled samples across the USA. The top panel is a bar chart showing the number of eggs used to make each genomic DNA preparation, in the middle is a bar chart showing the mapped read depth for each sample, and the lower charts show the frequency of resistance or susceptible alleles present at codons 198 and 200 of the isotype-1 β-tubulin gene. Blue bars indicate the susceptible alleles 198E(GAA) and 200F(TTC) at both the codons. (TIF) [file ppat.1011146.s009.tif]
