# Supplementary material for: Molecular evidence of widespread benzimidazole drug resistance in Ancylostoma caninum from domestic dogs throughout the USA and discovery of a novel β-tubulin benzimidazole resistance mutation
Source: PLoS Pathog. 2023 Mar 2;19(3):e1011146. doi: 10.1371/journal.ppat.1011146 (PMC10013918; doi:10.1371/journal.ppat.1011146)
Supplement: S1 Table — The table contains information on the 65 pooled samples that were used in the study, their geographical region, the number of samples in each pool, and the number of eggs that were used for genomic DNA preparation. (DOCX) [file ppat.1011146.s010.docx]

**S1 Table Information on pooled samples of *A. caninum***

The table contains information on the 65 pooled samples that were used in the study, their geographical region, number of samples in each pool, and the number of eggs that were used for genomic DNA preparation.

| **Pool** | **No. of Eggs** | **No. of Samples** | **Region** |
| --- | --- | --- | --- |
| P1 | 1000 | 13 | NE |
| P10 | 800 | 2 | NE |
| P11 | 300 | 4 | NE |
| P12 | 500 | 8 | NE |
| P13 | 300 | 7 | NE |
| P14 | 1000 | 6 | NE |
| P15 | 1500 | 8 | NE |
| P16 | 1100 | 7 | NE |
| P17 | 300 | 3 | NE |
| P18 | 1200 | 2 | NE |
| P19 | 1100 | 6 | NE |
| P2 | 2000 | 21 | NE |
| P20 | 1600 | 2 | NE |
| P21 | 1600 | 3 | NE |
| P22 | 1800 | 7 | NE |
| P23 | 600 | 6 | NE |
| P25 | 1225 | 7 | NE |
| P26 | 2700 | 11 | NE |
| P27 | 850 | 2 | NE |
| P29 | 575 | 2 | NE |
| P3 | 375 | 6 | NE |
| P30 | 550 | 3 | MW |
| P31 | 600 | 2 | MW |
| P32 | 1200 | 6 | MW |
| P33 | 1800 | 9 | MW |
| P34 | 1650 | 8 | MW |
| P35 | 5000 | 8 | MW |
| P36 | 6800 | 5 | MW |
| P37 | 1000 | 5 | MW |
| P38 | 1925 | 5 | MW |
| P39 | 2275 | 9 | MW |
| P4 | 1000 | 10 | NE |
| P40 | 6775 | 2 | MW |
| P41 | 10350 | 2 | MW |
| P42 | 3900 | 5 | MW |
| P43 | 200 | NA | MW |
| P44 | 325 | 2 | MW |
| P45 | 1000 | 3 | S |
| P46 | 2000 | 4 | S |
| P47 | 450 | 3 | S |
| P48 | 475 | 3 | S |
| P49 | 525 | 6 | S |
| P5 | 1000 | 4 | NE |
| P50 | 400 | 6 | S |
| P51 | 7425 | 3 | S |
| P52 | 2350 | 2 | S |
| P53 | 500 | 2 | S |
| P54 | 1550 | 2 | S |
| P55 | 1650 | 2 | S |
| P56 | 8000 | 2 | S |
| P57 | 1250 | 2 | W |
| P58 | 2950 | 2 | MW |
| P59 | 300 | 2 | W |
| P6 | 600 | 9 | NE |
| P60 | 450 | 2 | MW |
| P62 | 550 | 3 | MW |
| P63 | 1450 | 3 | S |
| P64 | 800 | 2 | S |
| P66 | 3500 | 2 | S |
| P67 | 3100 | 3 | W |
| P68 | 3500 | 12 | S |
| P69 | 2000 | 12 | S |
| P7 | 800 | 6 | NE |
| P8 | 1000 | 2 | NE |
| P9 | 1000 | 6 | NE |
