# Supplementary material for: Molecular evidence of widespread benzimidazole drug resistance in Ancylostoma caninum from domestic dogs throughout the USA and discovery of a novel β-tubulin benzimidazole resistance mutation
Source: PLoS Pathog. 2023 Mar 2;19(3):e1011146. doi: 10.1371/journal.ppat.1011146 (PMC10013918; doi:10.1371/journal.ppat.1011146)
Supplement: S2 Table — (DOCX) [file ppat.1011146.s011.docx]

**S2 Table: Primers used for Sanger sequencing of the near full-length isotype-1 β-tubulin**

| **Primer Name** | **Sequence** |
| --- | --- |
| AC_BT1_San_152_F1 | GGTGAATCTGATCTGCAACTT |
| AC_BT1_San_173_R5 | CAAGTTGCAGATCAGATTCAC |
| AC_BT1_San_389_F2 | CTTGATGTAGTTCGCAAAGAGG |
| AC_BT1_San_409_R4 | CTCTTTGCGAACTACATCAAGG |
| AC_BT1_San_577_F3 | GTGGAGCCATACAATGCTAC |
| AC_BT1_San_964_R3 | TCCAAGCGAGTGAGTCAATTG |
| AC_BT1_San_1051_R1 | GTTCTTGTTCTGCACTGACATC |
| AC_BT1_San_2320_R2 | GTGTAGCATTGTATGGCTCCA |
| AC_BT1_San_2963_F4 | GATGTCAGTGCAGAACAAGA |
