# Supplementary material for: Molecular evidence of widespread benzimidazole drug resistance in Ancylostoma caninum from domestic dogs throughout the USA and discovery of a novel β-tubulin benzimidazole resistance mutation
Source: PLoS Pathog. 2023 Mar 2;19(3):e1011146. doi: 10.1371/journal.ppat.1011146 (PMC10013918; doi:10.1371/journal.ppat.1011146)
Supplement: Attachment — Submitted filename: Reviewers_response_letter.pdf [file ppat.1011146.s014.pdf]

UNIVERSITY OF CALGARY

23rd December, 2022

Response to reviewer comments for the manuscript

Many thanks for your positive review of the first version of our submitted manuscript and for the reviewers' valuable and constructive comments. We are now submitting a revised version of the manuscript addressing the reviewers' comments and hope it is nowsuitable for publication in PLoS Pathogens.

Best regards,

Abhinaya Venkatesan and John Gilleard on behalf of the co-author team.

Our response to each of the reviewer's comment, along with the changes to the manuscript are itemized line-by-line below using the following scheme:

Reviewer comments are in Italic font.

Our responses are in blue font.

Changes to the manuscript in response to the comments are in red font.

Response to Reviewer 1:

The manuscript describes observations on benzimidazole anthelmintic resistant Ancylostoma caninum associated with point mutations in the β-tubulin gene and identify a previously unrecognized site, Q134. The observations are original, significant and are

anticipated to have an impact on the field of study. The authors should address the critiques before publication.

We thank Reviewer 1 for their positive comments as well as detailed and constructive feedback, and their recommendation for publication following major revisions.

Generally,

The benzimidazole anthelmintics, although similar, do have differences in their chemical structure but are assumed to be homogenous without showing any diversity for binding to different mutants of the isotype-1β-tubulin gene.

Response: When we perform *in vitro* parasite assays for drug resistance, our goal is not to replicate the precise *in vivo* effect as this depends on numerous other factors affecting drug pharmacokinetics and pharmacodynamics *in vivo*, which are largely determined by the location of the parasite in the animal, and interactions between the physiology of the treated animal and the specific chemical and structural properties of the drug. Rather, when using *in vitro* assays, we are examining diverse parasite isolates with the goal of discriminating drug-resistant and drug-susceptible isolates. It is common practice for the drug analog used in an *in vitro* assay to serve as a representative for measuring drug susceptibility across the entire drug class, and for that analog to be different from that used *in vivo* for treating animals. The choice of the analog for *in vitro* tests depends on practical factors and, in particular, which analog gives the best discrimination between resistant and susceptible isolates. A good example to illustrate this point is the Larval Development Assay (LDA) used to test for macrocyclic lactone resistance in ruminant gastrointestinal nematodes. Ivermectin aglycone is the analog of choice because it provides the greatest discrimination between susceptible and resistant nematodes in that

test, even though it has poor *in vivo* efficacy and thus was never developed as a therapeutic agent.

The specific reasons for the benzimidazole analogs used for the different tests and procedures in response to the referee's comments are given below.

Examples are:

1) The benzimidazole anthelmintics that were used for the treatment of the dogs are not specified;

Response: The *A. caninum* populations examined in this study were derived from hookworm-positive fecal samples from pet dogs sent to IDEXX laboratories from veterinary clinics across the country and there is no specific information available on anthelmintic treatment history of each individual animal. However, the reviewer raises a good point that a comment regarding the anthelmintic drugs routinely used in pet dogs in the USA would be helpful and so, we have inserted a sentence to provide that information in the introduction.

Manuscript change: The following sentence has been added to the bottom of page 3 in the introduction: "Treatment and control are dependent on the routine use of broad-spectrum anthelmintic drugs, the most commonly used in pet dogs in the USA being benzimidazoles (fenbendazole and febantel), tetrahydropyrimidines (pyrantel), and macrocyclic lactones (moxidectin and milbemycin)."

2) Thiabendazole is used for the Egg Hatch Assay (page 14);

Benzimidazoles have generally low aqueous solubility and so are difficult to use for *in vitro* tests. Thiabendazole is the standard benzimidazole analog used for the Egg Hatch Assay

(EHA) (Taylor et al. 2002) because, due to its higher solubility, it provides excellent *in vitro* responses that yield more consistent dose-responses, and a much higher level of discrimination, than those benzimidazole analogs commonly used as therapeutics (*e.g.*, fenbendazole, albendazole). Additionally, there is a large body of documented evidence that the *in vitro* dose-response using thiabendazole yields an excellent discrimination of benzimidazole susceptibility, with a strong correlation with the *in vivo* drug response of drugs like fenbendazole and albendazole in animals. In fact, the data we show in Fig 1 illustrate that the resistant *in vitro* phenotype parallels the presence of the mutations that confer resistance. Furthermore, in a recent publication by members of our group (Jimenez et al., 2021) using hookworm isolates with proven *in vivo* resistance to fenbendazole and/or albendazole, we demonstrate a significant correlation between the IC95 and beta-tubulin allele frequency, even before including the Q134H mutation (of which we were not yet aware).

3) Albendazole, which is not used in dogs, is used to assess resistance in the ean243 and ean244; nocodazole, which is an antineoplastic agent and not used as an anthelmintic is used for structural modeling of the A. caninum isotype-1 β-tubulin.

Response: Assessing resistance to the *ean243* and *ean244* mutants; the Andersen lab has published several studies that show (using the *C. elegans* model) that all of the known beta-tubulin alleles cause equivalent levels of resistance to albendazole and fenbendazole (Dilks 2020, Dilks 2021 - both cited in manuscript). In these highly replicated assays, albendazole elicits the most robust effect and it can be quantitatively tuned by altering concentrations. Regarding the structural modeling; nocodazole was used as a model for the therapeutically relevant BZs because it is the only analog with an experimentally resolved

structure bound to tubulin. Clinically relevant molecules from the same drug class, such as albendazole, mebendazole, and fenbendazole, vary only at the opposite end of the molecule to that interacting with the drug binding pocket and so are not predicted to vary with respect to the modeling of the drug-protein interaction.

4) The absence of the canonical codon 198 and 200 benzimidazole resistance mutations may relate to the use absence of benzimidazole anthelmintics used for large animals.

Response: This is not the case as the benzimidazole used in dogs and ruminants is largely the same. The most commonly used benzimidazole drugs in dogs are fenbendazole and febantel (which is a fenbendazole pro-drug). Fenbendazole is also the most commonly used benzimidazole drug in sheep and cattle and so there is no difference in the main drug analogs used.

Abstract

1. Briefly, define prevalences and overall frequency. The use of frequency is sometimes confusing in the manuscript.

Response: We have added two sentences in the material methods to describe the variant calling method and also added two sentences to define the terms "frequency" and "overall frequency. We have also removed the statement "often at high frequency" from the abstract.

Manuscript changes

Page 21: "Variant calling was performed by aligning the generated ASVs to the *A. caninum* isotype-1 β-tubulin reference sequence (Genbank Accession: DQ459314.1) using a global (Needleman-Wunsch) pairwise alignment algorithm without end gap penalties. Following

alignment, the ASVs were discarded if they were <180 bp or >350 bp long, or if they had a percentage identity <70% to the reference sequence, or if the ASVs had fewer than 200 reads in a sample, or if they were not present in two or more samples. This additional filtering ensures the removal of spurious sequences."

Abstract: Deep amplicon sequencing on *A. caninum* eggs from 685 hookworm positive pet dog fecal samples revealed that both mutations were widespread across the USA,~~ often at high frequency,~~ with prevalences of 49.7% (overall frequency 54.0%) and 31.1% (overall frequency 16.4%) for F167Y(TTC>TAC) and Q134H(CAA>CAT), respectively.

Introduction

1. 2nd paragraph: Comment on the prior use of vaccination against Ancylostoma caninum using irradiated l3 larvae e.g. Miller 1965, J Parasitology.

Response: We thank the reviewer for this comment. However, in the second paragraph of the Introduction, we are specifically discussing the reasons for high anthelmintic use, and consequently high drug selection pressure, in greyhound kennels and so we don't think there is direct relevance in commenting on an irradiated larval vaccine, which has not been used clinically for many decades, and therefore is largely of historical or immunological interest only. We would prefer not to include it as it would be a distraction to the point being made.

2. 3rd paragraph: Comment on any zoonotic concerns with the increase in observations from dog parks.

Response: We have included the following sentence in the second sentence of the introduction to better emphasize the zoonotic risk of this parasite.

Manuscript change: Page 3, Introduction first Paragraph: “In addition, this parasite is also of public health relevance due to its zoonotic potential in causing cutaneous larval migrans in humans [5], and patent infections in humans have also been reported in tropical regions [6,7]”.

3. 4th paragraph: Comment on benzimidazole resistance in fungi and C. elegans that has been associated with other amino acid mutations, even though it is commented on later in the discussion. Some examples include: A165, H6, E198 and F200 Minagawa, Cells 2021 and; C. elegans A185P, E69G, Q131L, S145F, M257L and D404N variants (Hahnel et al. 2018).

Response: This is a good suggestion and so we have added a number of additional sentences to the fourth paragraph.

Manuscript change: Page 5, last paragraph: “Additionally, several other amino acid substitutions in the *C. elegans ben-1* gene (Q131L, S145F, A185P, M257I, D404N, G104S, G142E, G142R, E198K, and R241H), not yet identified in parasites, have been shown to confer benzimidazole resistance [29,32]. Mutagenesis studies in fungi have also identified mutations conferring benomyl resistance at codons H6Y, Y50C, Q134L, A165V, E198K, F200Y, and M257 [33-36].”

Results

1. Page 7. Top: Were any other mutations associated with resistance in fungi or C. elegans found at even low frequencies with the deep amplicon sequencing?

Response: The amplicons that were deep sequenced span from codons 102 to 168 for the 293 bp fragment and from codons 186 to 261 for the 340 bp fragment. Only one other

non-synonymous mutation was detected in the whole data set: A D128N (GAC>AAC) which was detected at a low frequency in just two kenneled greyhound samples (frequency 13.5% in both the samples) and absent from the pet dog samples. This mutation has not been described as associated with resistance in fungi or nematodes.

Manuscript change: Page 12: “Only one other non-synonymous mutation was detected in the whole data set: a D128N(GAC>AAC) substitution that was detected at low frequency in just two out of the 70 kenneled greyhound samples (frequency 13.5% in both samples) but was not detected in any of the 314 pet dog samples. This mutation has not been described as associated with resistance in fungi or other nematodes and was not further investigated at this point due to its low occurrence and frequency.”.

2. Page 7, 2nd paragraph: Mapping with nocodazole, contact with methyl ester terminus relevant for albendazole, mebendazole, and fenbendazole but not thiabendazole that has a thiazole group present. How does the thiazole group of thiabendazole fit in the model? This is clinically relevant when it is used to diagnose benzimidazole resistance with the EHAs.

Our response: We aligned thiabendazole to nocodazole. The thiazole group fits well into the pocket and even forms a nice interaction with E198, which is a resistance site highlighted in the paper (see reviewer figure below).

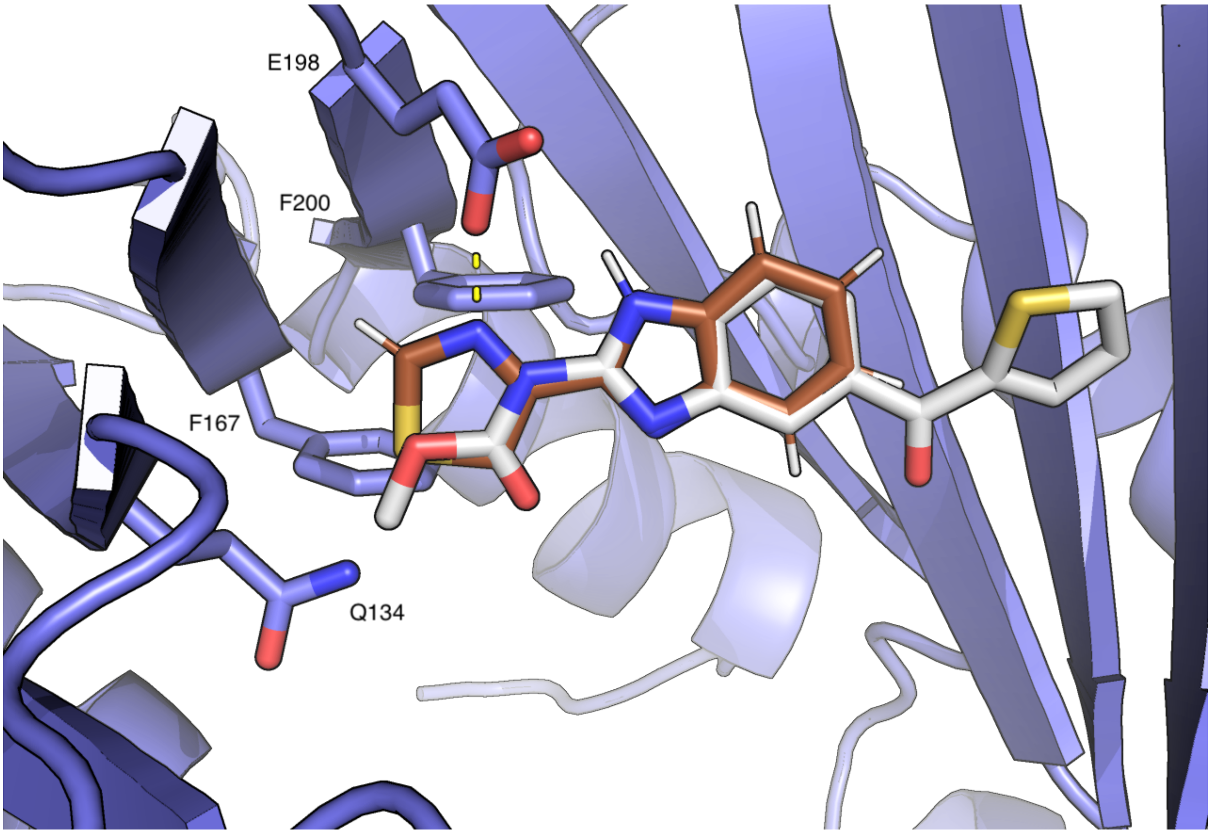

Manuscript change: We have included this figure in the supplementary information (S2 Fig):

"S2 Fig: **S2 Fig: *In-silico* protein structural model of the *A. caninum* isotype-1 β-tubulin bound to nocodazole with modeled thiabendazole**

Structural model for *A. caninum* isotype-1 β-tubulin made using AlphaFold2 (PMID: 34265844) (purple) aligned to Porcine β-tubulin (not shown) bound to nocodazole (white) (from PDB 5CA1) with thiabendazole modeled (brown). Potential interaction with residue 198 shown (dashed lines) with other features occupying the similar volume in the pocket to the methyl ester terminus."

3. Page 8, Top: Albendazole responses to the ean243 and ean244 suggest resistance. Do they show the same resistance to thiabendazole as well because thiabendazole is used in the EHAs (page 14) and the benzimidazole anthelmintics used for dogs?

Response: We only examined the response of the *C. elegans* allele-replacement strains to albendazole for the reasons given earlier in response to the reviewer's previous comment.

Discussion

1. Briefly comment if there is knowledge of dominance, recessive, homozygous, heterozygous and sex-linked effects of benzimidazole resistance and how this would affect spread.

Response: Whilst there is no specific knowledge in *A. caninum*, In the case of trichostrongylid nematodes of sheep, the benzimidazole resistance mutations are generally considered recessive (mainly homozygotes surviving *in vivo* drug treatment). However, this is a complex issue, as whether a resistance mutation behaves in a dominant or recessive fashion somewhat depends on the dose given, and is affected by other factors such as the pharmacokinetics of the drug. Given the complexity of the issue and the complete lack of evidence on the manner of inheritance for *A. caninum*, we think this issue is best avoided here.

2. Page 14: Not clear: ' …present in 99% (69/70) of A. caninum isolates sampled from greyhound……..and at high frequencies in most cases (> 50% in 62/70 isolates)…

Response: We have rephrased the sentence as follows:

Manuscript change: Page 15, second paragraph: "We previously reported that the canonical F167Y(TTC>TAC) isotype-1 β-tubulin benzimidazole resistance mutation was present in 99% (69/70) of *A. caninum* fecal egg samples from greyhounds in a number of racing and adoption kennels in the southern USA and at frequencies >50% in 62/70 samples."

Figures

Fig 1. Log IC95 values. Are the values -Log µM concentrations? What is the resistance threshold in µM?

Label Frequency as % ?

Response: The values are in Log µM concentration. The resistance threshold is 2.16 µM. For better clarity, we have modified the y-axis labels in Fig 1.

Manuscript change: new y-axis label for Fig 1A is "Log µM IC_{95}"

Fig 2. Thiabendazole Egg Hatch Assays were used for resistance estimations. More appropriate to fit thiabendazole than nocodazole which is an antineoplastic agent rather than an anthelmintic.

Response: As described earlier, nocodazole was used for *in silico* modeling as it is the only benzimidazole analog with an experimentally resolved structure bound to tubulin. Clinically relevant molecules from the same drug class, such as albendazole, mebendazole, and fenbendazole, vary only at the opposite end of the molecule to that interacting with the drug binding pocket and so are not predicted to vary with respect to the modeling of the drug-protein interaction.

Response to Reviewer 2:

This manuscript, entitled "Molecular evidence of widespread benzimidazole drug resistance in Ancylostoma caninum from domestic dogs throughout the USA and discovery of a novel β-tubulin benzimidazole resistance mutation" (PPATHOGENS-D-22-01783) examined pet dog hookworm populations from throughout the US for the presence of a know β-tubulin mutation (F167Y) that confers resistance to benzimidazole drugs. The authors used state of the art methodology to determine allele frequencies and showed that the allele has spread widely within the pet dog population. Perhaps more importantly, the authors identified a novel mutation (Q134H) associated with resistance and demonstrated that the homologous mutation introduced into C. elegans confers BZ resistance. While the data is robust, it is descriptive in nature, and some of the conclusions are unwarranted. Specifically:

1. Proclamation that this represents the first cases of drug resistant hookworms from pets is not accurate. Members of this group reported a miniature schnauzer (Tara) that was resistant to BZ as well as other anthelmintics (Jimenez Castro et al, 2019). Furthermore, while other reports of multidrug resistant hookworms were from greyhounds (Kitchen et al, 2019; Jimenez Castro et al 2019, 2021), these were rescued animals and considered pets. It was already clear that multidrug resistant hookworms have been slowly spreading into the pet population, and therefore it is disingenuous to claim this as the first report from pets.

2. Furthermore, it is a stretch to claim that drug resistance is "widespread" in the US based on the frequency of the F167Y allele alone. Given that phenotypic resistance requires the allele to be homozygous and is generally not detectable until it reaches at least 25% of the population, there are few places outside the West that have sufficiently high frequencies to conclude clinical resistance is present. What does seem apparent is that there are several

"hotspots" where resistant hookworms are likely to be common, such as CA, IL and New England. The author should tone down the claims of resistance, and instead use phrases like an increased likelihood of resistance or high frequencies of resistance alleles

Response: We respectfully disagree with several of the referee's comments. We did not claim in this manuscript that *"this is the first report from pets".* In fact, it was our own previous work that defined three clinical cases (Jimenez Castro 2019) along with another paper (Kitchen et al 2019). The focus of this submitted paper was on how widespread the benzimidazole resistance mutations in the pet dog population across the US, which was not known before, the use of amplicon sequencing in resistance surveillance and molecular epidemiology and the discovery and functional characterisation of a novel BZ resistance mutation.

For several reasons, we also respectfully disagree with the comment that "*it is a stretch to claim that drug resistance is "widespread" in the US based on the frequency of the F167Y allele alone. Given that phenotypic resistance requires the allele to be homozygous and is generally not detectable until it reaches at least 25% of the population, there are few places outside the West that have sufficiently high frequencies to conclude clinical resistance is present".* Firstly, the statement *"resistance is generally not detectable until it reaches at least 25% of the population"* dates back over 30 years (Martin P.J. et al., 1989) and is based on very limited evidence. Closer examination of this earlier work strongly suggests that this 25% figure is not accurate. Secondly, even if the 25% cutoff was true, it wouldn't change our statement: Our data show BZ resistance mutations are present in over half of all hookworm positive fecal samples in pet dogs from across the US, often at high frequency. In fact, the two resistance mutations are present at a combined frequency of

>50% in 117 / 393 samples (29.8%) of the pet samples analyzed. Consequently, we stand by our claim the data suggest widespread benzimidazole resistance in the US pet dog population.

Response to Reviewer 3:

I find exceptional merit in this manuscript. It has highly significant relevance for the potential of developing drug resistance in human hookworm populations as MDA campaigns intensify. The parasite under study is a zoonotic parasite (cutaneous larva migrans), which alone renders it suitable for this journal. The manuscript is well-written and concise. it blends an appealing mix of methods and the experiments lead to solid conclusions. The experimental design is sound, the figures clear and the conclusions fully justified. I can only congratulate the authors on their achievement.

Response: We thank the reviewer for their positive comments.

I have only two minor concerns. First, the last paragraph of the Introduction presents the results and conclusions of the work; it belongs, if anywhere, in the Discussion.

Response: We have deleted several sentences from the last paragraph of the introduction to simplify and just set the scene for the overall results.

Second, the authors should stress the zoonotic aspect of this parasite; while cases of CLM in the USA are rare, the increasing incidence of infection in companion animals and the inability to treat this MDR population is a public health threat and should be discussed.

Response: We have included some additional text to address this comment (see response to next comment below)

Manuscript change: Page 13, first paragraph: "Consequently, this widespread distribution of benzimidazole resistant *A. caninum* in pet dogs in the USA was not anticipated and and represents a major threat to sustainable control and management of clinical cases of canine hookworm infection in the USA which is highly dependent on anthelmintic drug use. In addition, benzimidazoles and ivermectin are used to treat clinical cases of cutaneous larval migrans in humans [38] and so, management of this zoonotic condition could also be compromised."

Finally, it behooves the authors to at least briefly discuss treatment options for MDR A. caninum in dogs and how to best diagnose this particular infection at this time. It is important to stress that the two main treatments for human CLM cases, albendazole and ivermectin, may be ineffective going forward.

Response:

Manuscript change: As discussed above , we have added the following sentences to the discussion at the top of page 13. "Consequently, this widespread distribution of benzimidazole resistant *A. caninum* in pet dogs in the USA was not anticipated and represents a major threat to sustainable control and management of clinical cases of canine hookworm infection in the USA which is highly dependent on anthelmintic drug use. In addition, benzimidazoles and ivermectin are used to treat clinical cases of cutaneous larval migrans in humans [38] and so, management of this zoonotic condition could also be compromised".

Response to additional reviewer comments:

Population allele frequency estimates based on bulk genotyping of eggs suffer from a number of biases (that are inherent in the sampling strategy). For this reason, it is important to provide a clear description of the parasite material used for the analysis. I suggest the authors use more specific descriptions such as "fecal egg samples" or "infrapopulations of (adult) parasites" instead of the term "isolates" to improve the clarity of the manuscript and to help readers better interpret the allele frequency data.

Response: We agree with the reviewer.

Manuscript change: We have changed the term "isolate" to "fecal egg samples" as suggested

Providing more details on amplicon sequencing (as supplementary material) would be helpful for readers to assess the data quality and potential technical problems that may confound the analysis. For each sample, please report the total number of sequenced reads and more importantly, the number of reads removed during each step of the quality filtering (base call quality, read length filter, DADA2 denoising step, read-merging, etc.).

Response: We have included two additional supplementary/ supporting tables (S3_Table, S4_Table) with information on the sequenced reads and the number of reads removed at each filtering step of the pipeline.

Manuscript changes:

Page 22, last paragraph: "The filtered reads from each step for the two amplicons are given in S3 and S4 Tables.:

Page 32

"S3 Table: Sequenced reads filtering for the 293 bp fragment encompassing codons 134 and 167

Information on the number of sequenced reads for each sample, the number of reads that were filtered during each step of the DADA2 pipeline and the variant calling pipeline for the 293 bp fragment.

S4 Table: Sequenced reads filtering for the 340 bp fragment encompassing codons 198 and 200

Information on the number of sequenced reads for each sample, the number of reads that were filtered during each step of the DADA2 pipeline and the variant calling pipeline for the 340 bp fragment."

Samples with a read depth of >1,000 were included in the analysis while a minimum depth of 200 was required for a sequence variant. For low-depth samples, this filtering scheme could result in false negative variant calls when variant allele frequency is below 20% (but higher than sequencing error rate). How was the value of this depth filter optimized?

Response: We chose these thresholds because, in our experience, trying to interpret samples with low read depths (<200 reads) leads to a risk of artifacts (due to contamination or barcode hopping). There actually were only six and three samples that passed QC but had read depths of <1000 for the 293 bp and 340 bp amplicons, respectively and so few samples were removed due to these thresholds. For samples with a total read depth only slightly greater than the 1000 read threshold, using a minimum ASV depth of 200 does carry the risk of missing low frequency mutations in those samples. However,

missing low frequency mutations in a few samples would not change any of our conclusions, and we believe this conservative approach is far preferable to including mutations that are artifacts.

The distribution of allele frequencies in fecal egg samples (e.g., Fig 4C) are not normally distributed. Furthermore, a bimodal distribution is observed in both the West and Northeast sample sets. The summary statistics used in the manuscript (e.g., CI and SEM) grossly underestimated standard errors and failed to effectively describe the observed distribution. It would be better to not use them as these statistics can be misleading.

Response: We agree with the reviewer. In response, we have removed the SEM column from the tables. For a more robust calculation of the 95% CI, we used the bootstrap method in R and have included the newer values in the tables, although they weren't very different from the original 95% CI values.

Manuscript changes:

Page 9, second paragraph: "The overall frequency in the positive samples was 54.0% (Bootstrap 95% C.I. 48.7% - 59.2%)"

Page 10, Table 1:

TABLE 1: PREVALENCE OF THE F167Y(TTC>TAC) MUTATION

**A: MEAN PREVALENCE OF F167Y ALLELE REGION WISE**					
**Region**	**Samples sequenced**	**Samples carrying 167Y allele**	**Mean allele frequency**	**Bootstrap 95% CI**	~~**Std error of mean (SEM)**~~
West	35	27	72.8	62.4, 82.7	~~10.7~~
Midwest	94	44	52.7	42.9, 62.2	~~10.1~~
South	92	44	40.2	30.9, 48.6	~~9.3~~
Northeast	93	42	57.8	46.6, 68.3	~~11.5~~
**B: MEAN PREVALENCE OF F167Y ALLELE BY BREED SIZE**					
**Breed size**	**Samples sequenced**	**Samples carrying 167Y allele**	**Mean allele frequency**	**Bootstrap 95% CI**	~~**Std error of mean (SEM)**~~

Small	50	18	45.5	29.2, 61.7	~~18.4~~
Medium	150	68	48.4	40.3, 56.3	~~8.1~~
Large	80	48	65.8	56.2, 74.7	~~9.4~~
**C: MEAN PREVALENCE OF F167Y ALLELE BY AGE OF THE DOG**					
**Age category**	**Samples sequenced**	**Samples carrying 167Y allele**	**Mean allele frequency**	**Bootstrap 95% CI**	~~**Std error of mean (SEM)**~~
Puppies (A)	61	36	49.7	39.8, 60.5	~~10.4~~
Young Adults (B)	85	42	50.3	39.3, 61.5	~~11.3~~
Mature Adults (C)	32	12	65.1	41.7, 84.5	~~24.3~~
Seniors (D)	28	7	63.6	34.7, 88.6	~~29.7~~

Page 10, first paragraph: "Its overall frequency in these positive samples was 16.4% (Bootstrap 95% C.I. 13.0% - 20.1%)"

Page 11, Table 2:

TABLE 2: PREVALENCE OF THE Q134H(CAA>CAT) MUTATION

**A: MEAN PREVALENCE OF Q134H ALLELE REGION WISE**					
**Region**	**Samples sequenced**	**Samples carrying 134H allele**	**Mean allele frequency**	**Bootstrap 95% CI**	~~**Std error of mean (SEM)**~~
West	35	18	15.1	7.4, 25.4	~~10.6~~
Midwest	94	20	23.7	15.2, 33.4	~~10.6~~
South	92	26	16.6	11.8, 21.7	~~5.3~~
Northeast	93	34	12.6	9.2, 16.8	~~3.9~~
**B:MEAN PREVALENCE OF Q134H ALLELE BY BREED SIZE**					
**Breed size**	**Samples Sequenced**	**Samples carrying 134H allele**	**Mean allele frequency**	**Bootstrap 95% CI**	~~**Std error of mean (SEM)**~~
Small	50	12	12.3	6.7, 19.1	~~7.5~~
Medium	150	36	17.6	12.7, 23.5	~~5.8~~
Large	80	38	11.0	8.7, 13.9	~~2.8~~
**C: MEAN PREVALENCE OF Q134H ALLELE BY AGE OF THE DOG**					
**Age category**	**Samples sequenced**	**Samples carrying 134H allele**	**Mean allele frequency**	**Bootstrap 95% CI**	~~**Std error of mean (SEM)**~~
Puppies (A)	61	20	18.7	10.5, 28.0	~~10.1~~
Young Adults (B)	85	27	14.9	9.7, 20.7	~~6.0~~
Mature Adults (C)	32	13	12.6	7.8, 18.1	~~6.1~~
Seniors (D)	28	5	9.3	2.8, 20.2	~~14.7~~

Page 12, first paragraph: “Its overall mean frequency in these positive samples was 25.8% (95% C.I. 20.1% - 31.0%)”

Page 23, first paragraph: “Bootstrap 95% confidence intervals for the mean resistance allele frequencies were calculated using the *boot* package in R [66].”

The expression “overall frequency” was used throughout the manuscript. Please consider using "mean allele frequency” instead. In addition, always clarify if the mean was computed across all samples or across only (positive) samples.

Response: We agree that clarification would be beneficial.

Manuscript Change: We have changed the term to “overall mean frequency in the positive samples” where appropriate

It appears that samples that did not pass the amplicon data QC were included in the main and supplementary figures. Please remove the failed samples before plotting. Throughout the manuscript, the term “histogram” was used to describe simple bar charts representing categorical data. These need to be corrected.

Manuscript change: We have removed the failed samples from the charts as requested and changed the term “histogram” to “bar chart” wherever appropriate throughout the manuscript.

I suggest revising the main figures, Figs 4 and 5 (and possibly combining the two). The upper panels in A showing the “number of eggs” and “read depth” are identical between the two figures and are redundant. In its currently format, it is difficult to examine the presence (or absence) of correlations between the read depth and allele frequency,

between the number of eggs and allele frequency, and between the F167Y allele frequency and Q134H allele frequency. Scatter plots of these variables would be more informative.

Response:. We have not combined figures 4 and 5 as it would affect the flow of the paper and we think the figure would be overcrowded. The read depth and egg count panels have been included simply to provide transparency to allow the reader to directly assess the quality and validity of the data. There is no expectation of a correlation between read depth, allele frequencies, and the numbers of eggs. Consequently, we have the figures in the current format.

In the absence of individual worm genotype data (as opposed to allele frequency data from eggs), it is difficult to examine the co-occurrence of Q134H and F167Y mutations in trans configuration. However, the co-occurrence of Q134H and F167Y mutations in cis configuration can be investigated using the sequencing data because the 293 bp amplicon encompasses both the codons 134 and 167. I wonder if the authors have looked for a double resistant recombinant haplotype in the dataset. These sequences may have been filtered out because the authors used DADA2 pipeline (which had been developed primarily for analyzing non-recombining microbial reads) that removes chimeric sequences. "Chimeric" reads are identified if they can be reconstructed by combining a left-segment and a right-segment from two more abundant "parent" sequences, and a recombinant haplotype would resemble a chimeric read.The authors hypothesized that the original selection for resistance occurred in greyhound kennels and the subsequent rehoming of retired greyhounds across the USA has led to the distribution of resistant A. caninum populations across a wide geographical range through environmental contamination. In addition to the Q134H and F167Y non-synonymous SNPs, the amplicons sequenced in this

study contain synonymous and intronic SNPs (that are closely linked to the resistance alleles). Analysis of the haplotype diversity using the amplicon data may provide additional insight into the origin and spread of these resistance alleles. Do Q134H and F167Y alleles occur on multiple distinct haplotype backgrounds that are geographically structured? Or are they primarily found only on one haplotype (despite the geographically broad sampling of worms)?

Response: These are all good points. However, we believe this is beyond the scope of this paper as more detailed genetic analysis is needed to provide meaningful insights into the origins and spread of resistance alleles. A separate paper is planned specifically looking at this question using molecular epidemiology and population genetics approaches including the analysis of a large dataset of neutral mitochondrial and nuclear markers.

Please check S2 Fig and S4 Fig for errors in the legends.

Response: We have checked the respective figure legends for any errors and are unable to find any.
